# Photo Quiz: Eccentric target sign in renal transplant recipient

**DOI:** 10.1128/jcm.00298-24

**Published:** 2024-10-16

**Authors:** Mahdi Ouafi, Caroline Couvreur, Arnaud Salmon-Rousseau, Anne-Sophie Deleplancque, Sarah Stabler, Claude-Alain Maurage, Camille Cordier

**Affiliations:** 1 Laboratory of Parasitology-Mycology, University Hospital of Lille, Lille, France; 2 Infectious Diseases Department, University Hospital of Lille, Lille, France; 3 Department of Pathology, University Hospital of Lille, Lille, France; 4 INSERM U1285, Unité de Glycobiologie Structurale et Fonctionnelle (CNRS UMR 8576), University of Lille, Lille, France; Mayo Clinic Minnesota, Rochester, Minnesota, USA

## PHOTO QUIZ 

The patient was a 55-year-old woman with a medical history of left facial palsy in 1989 with permanent sequelae, two consecutive renal transplantations in 1989 and 1991 for idiopathic extramembranous glomerulonephritis and a resection of an anastomotic false aneurysm of the iliac artery in 2005. Her immunosuppressive regimen consisted of mycophenolate acid at 720 mg twice a day, ciclosporin at 100 mg twice a day, and prednisolone at 5 mg/day. She worked in metallurgy and lived on a farm surrounded by a large number of cats.

**Fig 1:**
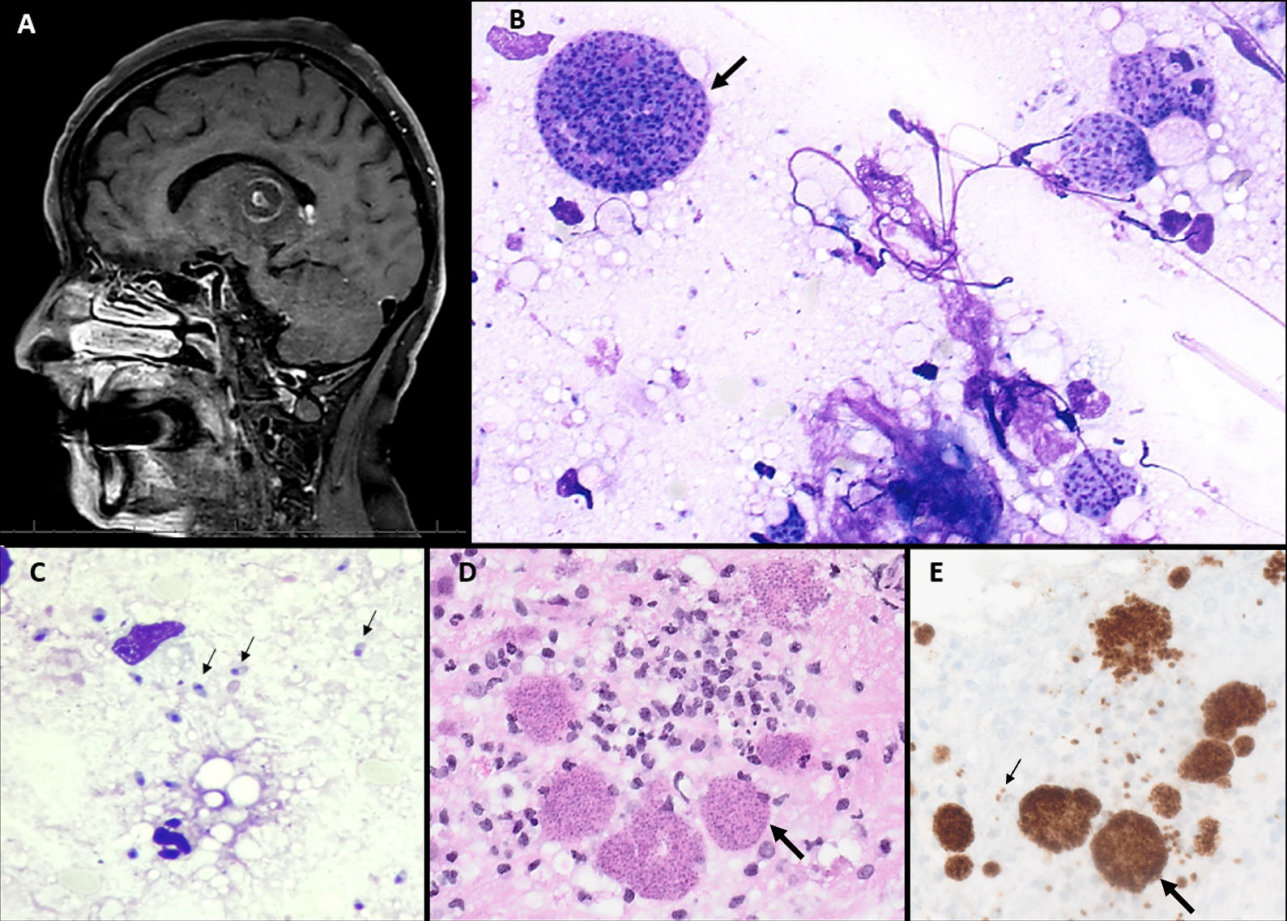
(**A**) Brain MRI T1 Fat Sat sequence injected with gadolinium, sagittal section. Nodular cystic lesion with ring enhancement and a small nodule (eccentric target sign). (B to E) Histology of the brain biopsy revealing cellular structures (indicated by black arrows) on Giemsa smears (**B and C**), hematoxylin-eosin-saffron staining (**D**), and strong immunoreactivity with specific antibodies (**E**).

Thirty-two years after the last renal transplantation the patient started to experience dizziness, clumsiness, left hemiparesis, and psychomotor slowing without fever. She was admitted 12 days later to the emergency room for an acute stroke suspicion for which brain magnetic resonance imaging (MRI) revealed a unique nodular cystic lesion measuring 23 × 19 mm with ring enhancement and a small eccentric nodule, known as the eccentric target sign, located in the right lentiform nucleus and thalamus associated with significant perilesional edema (Fig. 1A).

Laboratory tests showed anemia (9.4 g/dL), lymphopenia (0.3 × 10^9^ cells/L), elevated creatininemia (17 mg/L), and normal C-reactive protein levels (2.1 mg/L). A stereotactic brain biopsy of the lesion was performed 20 days later for suspicion of brain tumor or opportunistic infections. Giemsa, hematoxylin-eosin-saffron, and immunohistochemistry using specific antibody stainings were performed on the biopsy (Fig. 1B through E).

Despite treatment, the patient’s condition deteriorated, with the development of visual hallucinations, miosis, and then bilateral mydriasis. A cerebral computed tomography scan showed a 5 cm hematoma within the left thalamic lesion, causing quadri-ventricular flooding with acute hydrocephalus and diencephalic herniation.

What is your diagnosis?

## ANSWER TO PHOTO QUIZ

The patient was diagnosed with cerebral toxoplasmosis. The brain lesion biopsy revealed numerous cysts (Fig. 1B, D, and E, thick black arrows) and probable tachyzoite forms (Fig. 1C and E, thin black arrows) of *Toxoplasma gondii*. The parasite was identified based on its morphology. Bradyzoites are contained inside tissue cysts that vary in size and shape: early-stage cysts typically harbor a limited number of bradyzoites and measure as small as 5 µm in diameter. As they mature, the cysts incrementally enlarge up to 100 µm while bradyzoite divides through endodyogeny. Brain cysts commonly exhibit a rounded morphology. Tachyzoites are crescent-shaped, measuring approximately 2 by 6 µm, featuring a nucleus abundant in chromatin, with a pointed anterior end, and a rounded posterior end (1).

The presence of *T. gondii* was confirmed by immunohistochemistry using anti-*Toxoplasma gondii* antibodies (rabbit polyclonal antibody manufactured by Cell Marque, ref: 220A-15-ASR). *T. gondii REP529* gene-specific real-time polymerase chain reaction was positive on whole blood and brain biopsy. Serology revealed an increase in immunoglobulin G level and the presence of immunoglobulin A anti-*T*. *gondii*, confirming the toxoplasmosis reactivation.

*T. gondii* is a protozoan parasite and a member of the Apicomplexa phylum able to infect most warm-blooded animals. Humans are infected by ingesting undercooked meat containing cysts or oocysts-contaminated food/water (2). Other sources of contamination are congenital transmission, solid organ transplantation, and blood transfusion. While primary infections are in most situations benign for the immunocompetent, immunocompromised individuals are at risk of developing severe disseminated diseases during reactivation (3) or primary infection (4).

Cerebral toxoplasmosis can be responsible for multifocal lesions affecting various areas of the brain, but the « eccentric target sign » described with magnetic resonance imaging as a ring-shaped zone of peripheral enhancement with a small eccentric nodule along the wall can be found in less than 30% of cases but is considered pathognomonic with 95% specificity (5). The array of potential diagnoses for a ring-enhancing brain lesion notably encompasses a range of infectious diseases (including pyogenic bacterial abscess, tuberculoma, fungal abscess, and neurocysticercosis), malignancies, or demyelinating diseases (6).
